# Differences in adherence to COVID-19 pandemic containment measures: psychopathy traits, empathy, and sex

**DOI:** 10.1590/2237-6089-2020-0055

**Published:** 2020-09-22

**Authors:** Lucas de Francisco Carvalho, Gisele Magarotto Machado

**Affiliations:** 1 Universidade São Francisco CampinasSP Brazil Universidade São Francisco, Campinas, SP, Brazil.

The coronavirus disease 2019 (COVID-19) pandemic required the implementation of containment measures to slow the spread of the virus.^1 , 2^ The main measures are social distance, personal hygiene, and the use of face masks.^3^ Adherence to containment measures depends on individual factors,^4^ including personality traits.^5 , 6^ Previous evidence indicates that people with high levels of empathy tend to adhere more to containment measures.^7^ In contrast, people presenting high scores on the dark triad traits tend to adhere less to these measures.^8 , 9^ The expression dark triad refers to the set of three constellations of subclinical socially aversive traits: machiavellianism, narcissism, and psychopathy.^10^ Each component of the dark triad has specific characteristics, although they overlap in terms of manipulation and insensitivity traits.^11^ In this study, our focus was on one of the components of the dark triad, namely, psychopathy.

Psychopathy is characterized mainly by callousness and lack of empathy,^12^ including traits of irresponsibility, a tendency to behave in a socially deviant manner, tendency to deceive, grandiosity, recklessness, and impulsiveness.^13 , 14^ Moreover, psychopathy is associated with antisocial and criminal behaviors.^15 , 16^ Studies indicate that men score higher than women in psychopathy traits.^17 , 18^

Taking into consideration the association between individual differences and adherence to COVID-19 containment measures,^4^ as well as previous evidence indicating that typical psychopathic traits are associated with transgressive behaviors, this study aimed to investigate relationships between psychopathy traits and adherence to containment measures of the COVID-19 pandemic, also observing differences between men and women.

A total of 893 adult participants were included in the study. Age ranged from 18 to 79 years (mean [M] = 34.77; standard deviation [SD] = 11.98), they were mostly women (80%) and Caucasian (71.2%), and most reported having a graduate degree (39%).

Participants answered a web-based questionnaire released on online social networks containing questions about adherence to COVID-19 pandemic containment measures, facets of the Personality Inventory for DSM-5 (PID-5),^19^ which assesses pathological personality traits, and the Affective and Cognitive Measure of Empathy (ACME),^20^ which evaluates the empathy trait through the affective resonance indicator. Regarding adherence indicators, the items focused on four dimensions: social distancing (engagement to social distance measure; three items), hygiene (engagement in hygienic recommendations; three items), face mask (using face mask; two items), and staying home (never leaving home; one item).

After approval by the Universidade São Francisco research ethics committee, data collection was performed online using Google Forms. We shared the survey link on the social media website Facebook and also via the WhatsApp application, inviting individuals to participate and relying on the snowball strategy to reach a larger number of participants.

We used latent profile analysis to empirically discriminate groups according to the scores obtained on the personality measures. Latent profile analysis is recommended to the investigation of different subpopulations, according to distinct answer patterns to a group of variables.^21 , 22^ For this analysis, we used the following indicators: scores on affective resonance (ACME), callousness, deceitfulness, grandiosity, impulsivity, irresponsibility, and risk-taking (PID-5). Previously to this analysis, we standardized the scores in *z* (M = 0; SD = 1). Comparisons between means were conducted using analysis of variance (ANOVA) to assess differences in adherence to the containment measures, including the groups identified by the latent profile analysis and the sex variable. For ANOVA, we used 0.05 as significance level, and the partial eta squared was used as an effect size indicator. The partial eta squared was interpreted as 0.01 (small), 0.09 (medium) and 0.25 (large).^23^ Latent profile analyses were performed in the software Mplus version 7, and ANOVA in the Statistical Package for the Social Sciences (SPSS) version 23.

**Figure 1:**
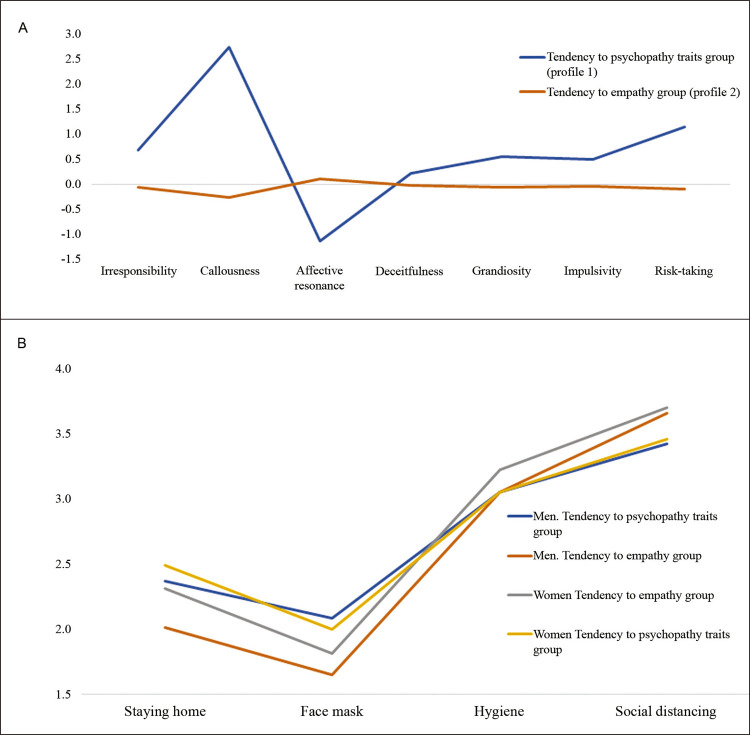
A) Composition of profiles obtained via latent profile analysis; B) means obtained according to profile and sex.

For the latent profile analysis, we tested solutions with two, three, and four profiles. Although the two-profile solution did not demonstrate the best-fit indices, it did have acceptable fit indices,^24 - 26^ and better interpretability for the observed profiles. The adjustment indices were adjusted Bayesian information criteria (aBIC) = 16883.167; entropy = 0.978; Lo-Mendell-Rubin adjusted likelihood ratio test (LMRT) = 0.0001. Figure 1A shows the two profiles observed.

The tendency to psychopathy traits group (profile 1) comprised 77 participants who showed higher means in psychopathy traits and lower affective resonance; 7.4% of the all the participating women and 12.8% of all the men were in this group. The tendency to empathy group (profile 2) comprised 816 people, and had a higher level of affective resonance and lower scores on psychopathy traits; 92.6% of women and 87.2% of men were in this group.

The groups found in the latent profile analysis were compared in terms of adherence to containment measures against the COVID-19 pandemic. The sex variable was also considered in the analysis. Profiles differed significantly regarding adherence to containment measures, except in the hygiene dimension. There were no significant differences regarding sex. The results are shown in Table 1 .

**Table 1:** Profile and gender comparison regarding adherence to containment measures.

Group/variable	Sum of squares	df	F	p	Partial η2
Sex					
Stay home	2.51	1.00	2.16	0.14	0.00
Social distancing	0.11	1.00	0.41	0.52	0.00
Hygiene	0.42	1.00	0.96	0.33	0.00
Face mask	0.09	1.00	0.08	0.78	0.00
Profile					
Stay home	4.47	1.00	3.85	0.05	0.01*
Social distancing	3.45	1.00	12.65	0.00	0.01*
Hygiene	0.45	1.00	1.03	0.31	0.00
Face mask	5.61	1.00	5.20	0.02	0.01*
Sex*profile					
Stay home	0.49	1.00	0.42	0.52	0.00
Social distancing	0.00	1.00	0.00	0.98	0.00
Hygiene	0.47	1.00	1.08	0.30	0.00
Face mask	0.86	1.00	0.80	0.37	0.00
Error					
Stay home	1032.13	889.00			
Social distancing	242.11	889.00			
Hygiene	390.39	889.00			
Face mask	959.82	889.00			

* Small partial η2.

This study aimed to investigate relationships between indicators of adherence to COVID-19 containment measures and indicators of psychopathy. In addition, we assessed the impact of the sex variable on that relationship. The results indicated that people with increased psychopathy traits and low levels of empathy tend to adhere less to containment measures in comparison to people not showing these characteristics, which is in line with previous findings suggesting personality traits as associated with adherence to containment measures in the COVID-19 pandemic.^5 - 6 , 8 - 9^ Furthermore, our findings add to the existing literature^7 , 15 - 16^ by indicating traits of psychopathy as associated with transgressive behaviors, and empathy traits as associated with cooperation. Conversely, even though there is evidence suggesting that men are more likely to exhibit behaviors typical of psychopathy than women,^17 , 18^ no significant differences were found regarding adherence to containment measures and sex. These findings may be related to the manifestation of psychopathy traits in men and women. For instance, male psychopaths often manifest impulsivity and conduct problems such as violent behavior, whereas female psychopaths usually engage in running away, self-harming behaviors, manipulation, and property crimes such as theft or fraud.^27^

Our findings indicate that psychopathy traits should be accounted for as relevant while establishing public policies to increase and maintain adherence to COVID-19 containment measures. These findings should be considered for both men and women, as we did not observe differences regarding sex.

The present findings should be considered in light of the methodological limitations of our study. First, the data were collected online, which may imply a bias regarding the demographic characteristics of the sample. Second, the sample consisted of a larger number of women (80%), which may have skewed the findings. Given these limitations, we recommend that this study be replicated using representative samples. We also suggest that other studies investigate the interaction between psychopathic traits and other variables and their influence on adherence to containment measures.
